# The Role of Noncoding RNA Pseudouridylation in Nuclear Gene Expression Events

**DOI:** 10.3389/fbioe.2018.00008

**Published:** 2018-02-08

**Authors:** Yang Zhao, William Dunker, Yi-Tao Yu, John Karijolich

**Affiliations:** ^1^Department of Pathology, Microbiology, and Immunology, School of Medicine, Vanderbilt University, Nashville, TN, United States; ^2^Department of Biochemistry and Biophysics, Center for RNA Biology, School of Medicine and Dentistry, University of Rochester, Rochester, NY, United States; ^3^Vanderbilt-Ingram Cancer Center, Nashville, TN, United States

**Keywords:** RNA pseudouridylation, steroid receptor RNA activator, 7SK RNA, spliceosomal small nuclear RNA, transcription, pre-mRNA splicing

## Abstract

Pseudouridine is the most abundant internal RNA modification in stable noncoding RNAs (ncRNAs). It can be catalyzed by both RNA-dependent and RNA-independent mechanisms. Pseudouridylation impacts both the biochemical and biophysical properties of RNAs and thus influences RNA-mediated cellular processes. The investigation of nuclear-ncRNA pseudouridylation has demonstrated that it is critical for the proper control of multiple stages of gene expression regulation. Here, we review how nuclear-ncRNA pseudouridylation contributes to transcriptional regulation and pre-mRNA splicing.

## Introduction

The proper control of gene expression in the nucleus is achieved by the actions of a diverse set of factors. In addition to proteins, noncoding RNAs (ncRNAs) participate in most, if not all, stages of nuclear gene expression, including both RNA polymerase II (Pol II) transcription and pre-mRNA splicing (Mercer et al., 2009). ncRNAs employ numerous mechanisms to accomplish their function, including binding to and modulating protein function, base pairing with complementary nucleic acids, or directly catalyzing biochemical reactions. Like proteins, proper modification and folding into higher-order structures are a prerequisite for their function.

**Figure 1:**
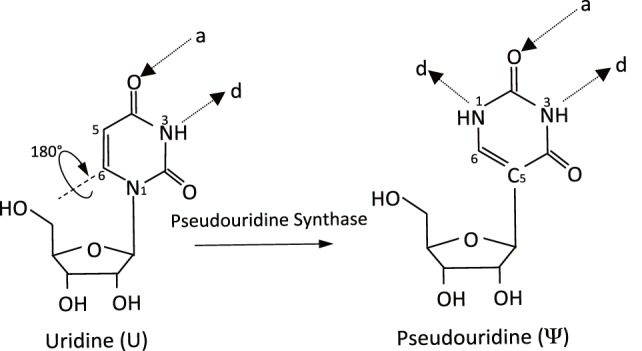
Schematic of the pseudouridylation reaction. The isomerization of uridine (U) to pseudouridine (ψ) is mediated by pseudouridine synthases (PUSs). It results in an extra hydrogen bond donor (d) and the same number of hydrogen bond acceptors (a).

In addition to the four canonical nucleosides, more than 140 chemically distinct modified RNA nucleosides have been identified in nature (Machnicka et al., 2013). Pseudouridine (ψ), first discovered over 60 years ago (Cohn and Elliot, 1951), is the most abundant internal RNA modification in stable RNAs. ψ, the C5-glycoside isomer of uridine, is formed through an internal transglycosylation reaction in which the N1–C1' bond between the uracil base and the ribose sugar is broken and a C5–C1' glycosidic bond is reformed (Figure 1). As a consequence of isomerization, an additional hydrogen bond donor is present at the non-Watson–Crick edge. The distinct structure of ψ increases both the rigidity of the phosphodiester backbone and the thermodynamic stability of ψ–A compared with U–A. This effect is mediated by water-coordinated hydrogen bonding and base stacking (Charette and Gray, 2000).

Initial evidence for a functional role of ψ partially came from the fact that ψ residues are clustered in functionally important and evolutionarily conserved regions of tRNA (Grosjean et al., 1995; Hopper and Phizicky, 2003), rRNA (Branlant et al., 1981; Maden, 1990), and small nuclear RNA (snRNA) (Reddy and Busch, 1988; Massenet et al., 1998; Narlikar et al., 2002; Karijolich and Yu, 2010). Indeed, experimental data have confirmed important roles for pseudouridylation in multiple aspects of gene expression regulation, including spliceosomal small nuclear ribonucleoprotein (snRNP) biogenesis, efficiency of pre-mRNA splicing, and translation fidelity (Karijolich et al., 2010). Here, we will provide an overview of the mechanisms of pseudouridylation and then highlight several pseudouridylated ncRNAs and their effects on nuclear gene expression events, specifically transcription and pre-mRNA splicing.

## Mechanisms of Pseudouridylation

The past decades have seen remarkable progress toward defining mechanisms by which pseudouridylation is catalyzed. Pseudouridylation of ncRNA is catalyzed by pseudouridine synthases (PUSs) through two distinct mechanisms, namely RNA-dependent pseudouridylation and RNA-independent pseudouridylation.

## RNA-Dependent Pseudouridylation

**Figure 2:**
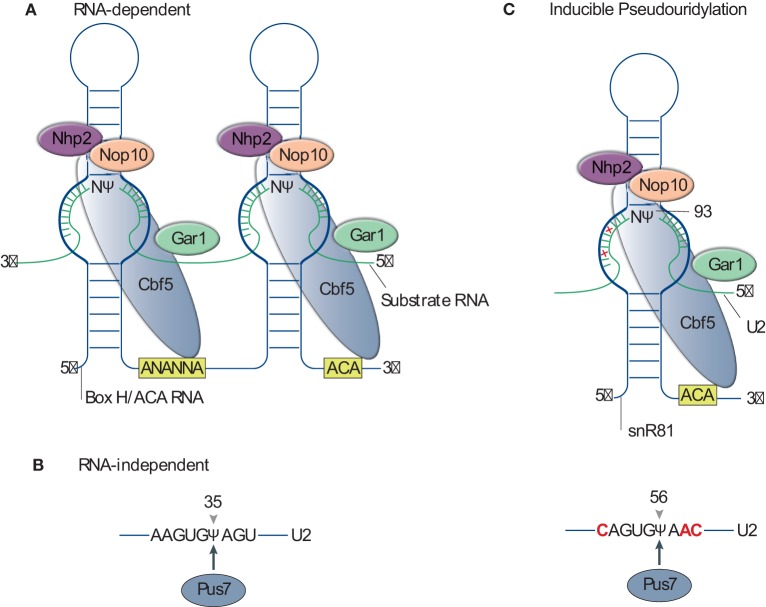
Mechanism of pseudouridylation. **(A)** Schematic of RNA-dependent pseudouridylation by box H/ACA RNP. The box H/ACA RNP is composed of the box H/ACA RNA and four core proteins (Cbf5, Nhp2, Nop10, and Gar1). The secondary structure of a eukaryotic pseudouridylation guide box H/ACA RNA is shown as a blue line. The RNA adopts a hairpin-hinge–hairpin-tail structure. The box H (5'-ANANNA-3') within the hinge region and the box ACA (5'-ACA-3') motif at the 3'-end of the RNA are highlighted in a yellow box. The pseudouridylation pocket (thick-blue line) facilitates substrate recognition *via* complementary base-pairing interactions between the box H/ACA RNA and the substrate RNA (green line). **(B)** The standalone pseudouridine synthase (PUS), PUS7, recognizes the consensus sequence of substrates U2 small nuclear RNA (snRNA) and catalyzes the pseudouridine formation at U35. **(C)** Inducible pseudouridylation of U2 snRNA. Top, the 3' pocket of small nucleolar RNA 81 (snR81) base pairs with the sequence surrounding nucleotide (nt) U93 with two mismatches (denoted as red crosses). Bottom, the sequence surrounding U56 recognized by PUS7 has three nts (highlighted in red), different from the consensus PUS7 recognition sequence.

The RNA-dependent pseudouridylation machinery consists of one unique box H/ACA RNA and four core proteins. Box H/ACA RNAs are one of the most evolutionarily conserved families of small ncRNAs and are present in all eukaryotes. They function as guide RNAs to direct pseudouridylation in mRNA, rRNA, spliceosomal snRNAs, and various other types of ncRNAs. With a median length of 133 nucleotides (nts), eukaryotic box H/ACA RNAs adopt a hairpin-hinge–hairpin-tail secondary structure (Figure 2A). Within the internal hinge region, there is a H box motif (ANANNA), while an ACA box motif (ACA) is positioned near the 3' end. Within each hairpin structure, an internal loop (pseudouridylation pocket) is present, which facilitates substrate recognition *via* complementary base-pairing interactions between the box H/ACA RNA (the loop sequence) and the substrate RNA. Both hairpins of H/ACA RNAs can carry functional pseudouridylation pockets and can thus independently direct pseudouridylation of uridines in separate RNAs or separate uridines located within the same substrate RNA. In the guide RNA–target RNA interaction, the guide sequences hybridize 5' and one nt 3' of the target uridine to the substrate RNA immediately, thereby framing it. The distance between the target uridine and the H or ACA box of the guide RNA is usually 14–15 nts (Kiss et al., 2010).

The four core proteins associated with box H/ACA RNAs are Cbf5 (dyskerin in human and NAP57 in rodents), Nhp2 (L7Ae in archaeal), Gar1, and Nop10 (Yu and Meier, 2014). Cbf5 is the enzymatic component of the RNP and catalyzes the U-to-ψ isomerization reaction (Figure 2A). Structures of the enzymes from various species show a high degree of evolutionary conservation, especially in the PUS and Archaeosine transglycosylase (PUA) domain (Hamma et al., 2005; Manival et al., 2006; Rashid et al., 2006). The other three core proteins are also essential, and the depletion of them in yeast, with the exception of GAR1, causes the loss of all H/ACA RNAs (Girard et al., 1992; Bousquet-Antonelli et al., 1997).

Facilitated by the development of *in vitro* systems for the reconstitution of enzymatically active RNP complexes, the crystal structure of the box H/ACA RNP was first solved using archaeal components (Li and Ye, 2006). These experiments demonstrated that the L7Ae, Nop10, and the catalytic domain of Cbf5 bound to the upper stem of the guide RNA, whereas the PUA domain of Cbf5 anchored the lower stem and ACA motif. The substrate RNA is recruited and the target uridine is precisely placed within the active site *via* complementary base-pairing interactions with the bipartite guides, while extensive protein interactions help to stabilize the interaction. In contrast to the other core proteins, Gar1 does not physically interact with the box H/ACA guide RNA or substrate RNA. Instead, Gar1 interacts directly with the thumb loop of Cbf5 and participates in regulating substrate turnover. Studies in yeast have revealed that the structure of the eukaryotic box H/ACA RNP is highly similar to the archaeal one, however, with exceptions. In particular is the independence of the RNP’s activity from Nhp2 binding and a novel C-terminal extension in Gar1, which interacts with Cbf5. It is hypothesized that these functional and structural differences reflect the evolutionary adaptations of eukaryotic box H/ACA RNP to the variable RNA structure and moderate temperature range in which eukaryotes live in, respectively (Li et al., 2011).

## RNA-Independent Pseudouridylation

RNA-independent pseudouridylation in eukaryotes acts through standalone enzymes called PUS enzymes. In contrast to the box H/ACA RNP-based mechanism, PUS enzymes carry out both substrate recognition and the internal transglycosylation reaction. Substrate recognition is achieved *via* consensus sequences and/or secondary structure elements of the substrate RNA (Figure 2B). In eukaryotes, there are 10 different PUS enzymes, numbered PUS1 through PUS10. These are classified into five families (TruA, TruB, TruD, RluA, and PUS10) based on their bacterial counterparts (Hamma and Ferre-D’Amare, 2006). Although the primary sequences have diverged, all PUSs, including Cbf5, share a conserved catalytic domain and likely a conserved catalytic mechanism based on the solved crystal structure (Foster et al., 2000; Hoang and Ferre-D’Amare, 2001, 2004; Sivaraman et al., 2002, 2004; Del Campo et al., 2004; Ericsson et al., 2004; Kaya et al., 2004; Mizutani et al., 2004; Hoang et al., 2006; McCleverty et al., 2007). This domain structure is composed predominately of anti-parallel β-sheets, with one face decorated by two groups of α-helices and loops. A forefinger–thumb structure formed by these loops pinches the target RNA, while the strictly conserved catalytic aspartate residue participates in the enzymatic reaction.

Unlike the box H/ACA RNPs, which have been found only to reside within the nucleus, PUS enzymes have been found in the nucleus, cytoplasm, and mitochondria. Each PUS enzyme targets either one specific or multiple uridines in many RNA species, including snRNAs, rRNAs, and tRNAs in both cytoplasm and mitochondria.

## Pseudouridylation is Inducible

Until relatively recently, RNA modifications, including pseudouridylation, were considered constitutive. In 2011, Wu et al. (2011) provided the first evidence that RNA modifications were inducible and demonstrated that yeast U2 snRNA was conditionally pseudouridylated when cells were subjected to nutrient deprivation or heat shock (Wu et al., 2011). In addition to the three constitutive pseudouridines (ψ35, ψ42, and ψ44) of yeast U2 snRNA, two novel pseudouridines (ψ56 and ψ93) were detected in stressed cells. Further detailed analyses revealed that both the RNA-independent PUS (PUS7 catalyzes ψ56 formation) and the box H/ACA RNP-dependent [small nucleolar RNA 81 (snR81)-guided box H/ACA RNP catalyzes ψ93 formation] modification machineries are involved in inducible pseudouridylation. Interestingly, both ψ56 and ψ93 are “imperfect” substrates for their respective modification machineries. The sequences flanking positions U56 and U93 in U2 snRNA are similar, but not identical to the sequences surrounding the constitutively pseudouridylated targets of PUS7 and snR81, respectively. For example, the 3' pocket of snR81 base pairs with the sequence surrounding nt U93 of U2 snRNA with two mismatches. In addition, ψ56 formation is mediated by PUS7 engaging a substrate whose sequence differs by three nts from the consensus PUS7 recognition sequence (Figure 2C). Inducible pseudouridylation is also functionally relevant, as demonstrated by the observation that the artificial introduction of ψ93 reduces the efficiency of pre-mRNA splicing. Interestingly, it was recently shown that the TOR-signaling pathway regulates ψ93 formation (Wu et al., 2016b).

Inducible pseudouridylation of other snRNAs has been reported subsequently. U6 snRNA is inducibly pseudouridylated at U28 by PUS1 during the yeast filamentous growth program (Basak and Query, 2014). Further analysis of mutants indicates that U6–ψ28 is functionally relevant, as all U6 snRNA mutations that resulted in strong pseudouridylation at position U28 also exhibited a pseudohyphal growth phenotype, whereas blocking U6–ψ28 formation prevents filamentous growth.

Recently, transcriptome-wide mapping of pseudouridines in yeast and human cells has revealed its presence in mRNA (Carlile et al., 2014; Lovejoy et al., 2014; Schwartz et al., 2014). While mRNA pseudouridylation appears to be primarily catalyzed by the standalone PUSs (PUS1–PUS4, PUS6, PUS7, and PUS9), several pseudouridine residues are catalyzed by box H/ACA RNPs. Remarkably, mRNA pseudouridylation was also found to be highly inducible and in a stress-specific manner. For example, by comparing mRNA ψ profiles of untreated cells to those of cells exposed to heat shock or H_2_O_2_, it was found that the inducible pseudouridylation profiles were largely nonoverlapping (Li et al., 2015).

## Function of ncRNA Pseudouridylation in Transcription

**Figure 3:**
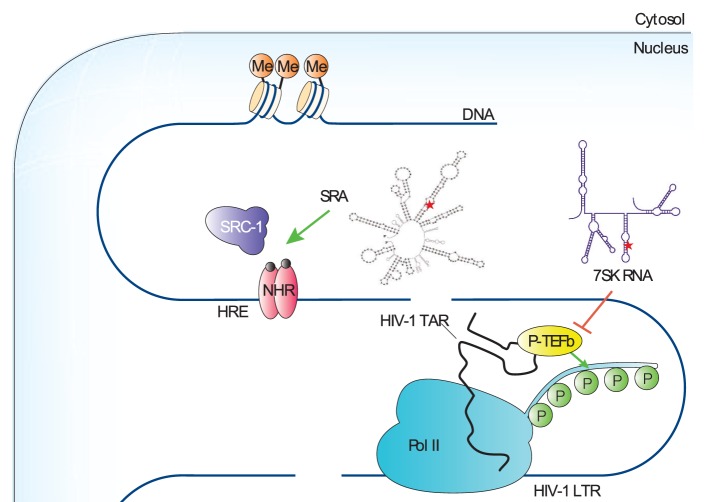
Functions of noncoding RNA (ncRNA) pseudouridylation during transcription. The secondary structures of ncRNAs are shown, and the pseudouridylation sites are denoted as a red star. The green arrow and red-blocking arrow highlight the ncRNAs that are known to regulate polymerase II (Pol II) transcription, the factors they target and whether the effect of the ncRNA on transcription is stimulatory or inhibitory, respectively. HIV-1 LTR, human immunodeficiency virus-1 long terminal repeat; HRE, hormone response element; HIV-1 TAR, human immunodeficiency virus-1 transactivation response; NHR, nuclear hormone receptor; P, phosphoryl group; P-TEFb, positive transcription–elongation factor-b; SRA; steroid receptor RNA activator; SRC-1, steroid receptor activator-1.

Transcription is the primary control point for gene expression. It therefore determines cellular function and cell identity and is subjected to tight regulation to achieve a high degree of specificity and efficiency. The eukaryotic DNA template is packaged by histone proteins into a highly condensed structure called chromatin. The chromatin structure is dynamically regulated by both histone modifications and chromatin-remodeling factors (Narlikar et al., 2002). Promoters contain elements that bind to transcriptional activators and repressors, as well as the transcription machinery (Smale and Kadonaga, 2003; Kadonaga, 2004). RNA Pol II is the enzyme to catalyze the transcription reaction of mRNA from DNA. Pol II is recruited to promoters by transcriptional activators in a holoenzyme form together with general transcription factors and a multiprotein complex called the Srb/Mediator (Bjorklund and Kim, 1996). Following transcription initiation, Pol II transits to a productive elongation status through interactions with multiple elongation factors (Zhou et al., 2012). Given the central role of transcription in gene expression, it is not surprising that transcription is subject to diverse steps of regulation. Here, we will discuss two pseudouridylated ncRNAs and their function in regulating RNA Pol II transcription (Figure 3).

## Steroid Receptor RNA Activator (SRA) and Transcription Preinitiation

One layer of transcriptional control comes from the binding of activator and repressor proteins to the promoters of target genes in a sequence-specific manner (Smale and Kadonaga, 2003). Coactivators and corepressors, which interact with activators and repressors, are required to achieve optimal transcriptional regulation in cells (Kadonaga, 2004). The SRA was first identified as a transcriptional coactivator for several steroid-hormone receptors, including receptors for androgens (ARs), estrogens (ERs), glucocorticoids (GRs), and progestins (PRs) (Lanz et al., 1999). Interestingly, while it was initially presumed that a protein encoded by a specific 5'-spliced variant of the SRA gene was the functional factor, subsequent experiments demonstrated that the factor was an ncRNA.

Steroid receptor RNA activator operates as part of a ribonucleoprotein complex containing steroid receptor activator-1, which is an AF-2 coactivator (Lanz et al., 1999). Computer-assisted modeling suggests that SRA adopts a highly complex secondary structure containing 11 topological substructures (STRs). Mutagenesis of each STR indicated that 5 of 11 STRs are required for SRA to coactivate transcription, and STR7 is the most important one for SRA function (Lanz et al., 2002).

In a study to identify coactivators for retinoic acid receptor (RAR) in mouse S91 melanoma cells, mPUS1p was unexpectedly identified. In addition, SRA turned out to be a substrate of mPUS1p (Zhao et al., 2004). Using chromatin immunoprecipitation, RAR, PUS1p, and SRA were found to cooccupy the retinoic acid response promoter in a ligand-independent complex. PUS1-mediated pseudouridylation of SRA promotes the formation of an “active” structure and aids in establishing the transcription preinitiation complex upon ligand binding. Further supporting a role of SRA pseudouridylation in transcriptional regulation, mutations in PUS1p that disrupt its interaction with RAR or its pseudouridylation activity attenuate the activation of RAR-dependent transcription. mPUS1p also significantly augmented transactivation by other nuclear receptors (NRs) including thyroid hormone receptor (TR), GR, AR, PR, and ER, illustrating that this mechanism likely applies universally to the regulation of NR-dependent transcription.

In addition to mPUS1p, mPUS3p also modifies SRA and serves as an NR coactivator (Zhao et al., 2007). Unlike mPUS1p, mPUS3p does not enhance sex steroid receptor activity, suggesting that substrate-site specificity may have distinct roles. Indeed, *in vitro* mPUS1p and mPUS3p generally modify different positions in SRA, with a few positions commonly targeted. Intriguingly, the order of modification of SRA by mPUS1p and mPUS3p determines the positions within SRA that are required to be pseudouridylated. However, it is important to note that the only *in vivo*-pseudouridylated site identified in SRA is U206. Interestingly, a U206A mutation, which promotes hyperpseudouridylation of SRA *in vitro*, switches SRA from a coactivator to a molecule with dominant-negative activity *in vivo*.

Pseudouridylation of SRA by mPUS1p and mPUS3p is a highly complex posttranscriptional mechanism that controls a coactivator–corepressor switch in SRA with major consequences for NR signaling (Zhao et al., 2007). Unexpectedly, pseudouridylation of SRA occurs in a stem-loop structure STR5 (at position U206), whose secondary structure was not shown to be important for SRA function. Moreover, the thermodynamic and secondary structure differences between STR5 in hSRA-WT and hSRA-U206A are relatively minor, which further suggest that the secondary structure remodeling is unlikely to explain the large biochemical and functional effects observed. Instead, it is proposed that ψ206 stabilizes stems I and II of STR5 in a higher-order conformation through the base-stacking-enhancing properties of ψ, resulting in masking new sites and preventing hyperpseudouridylation by mPUS1p and mPUS3p. This in turn may interfere with the binding of SRA to other proteins that define its function as a scaffold for both repressors and activators.

The physiological importance of PUS1p-mediated SRA pseudouridylation is illustrated by a disorder known as mitochondrial myopathy and sideroblastic anemia (MLASA). It is caused by an inactivating mutation in human PUS1p (Bykhovskaya et al., 2004). Some abnormalities in these patients, such as facial dysmorphisms, are suggested to be the consequence of defective hSRA–NR signaling (Fernandez-Vizarra et al., 2007). In addition, ERs and ARs are important targets for cancer therapy. Given the importance of SRA in NR-transcriptional regulation, coupled with the functional significance of PUS1p-mediated SRA pseudouridylation, recent work has suggested that the disruption of SRA pseudouridylation could serve as a novel RNA-based cancer therapeutic (Ghosh et al., 2012).

## 7SK snRNA and Transcription Elongation

For the past three decades, most of the attention has been put on the early stages of the transcription cycle involving the recruitment of Pol II to gene promoters and assembly of active preinitiation complexes, which were thought to be the principal points where transcription was controlled (Kuras and Struhl, 1999; Ptashne, 2005). In recent years, however, accumulating evidences indicate that the subsequent stages of the transcription cycle are also highly regulated (Guenther et al., 2007; Muse et al., 2007). Notably, the promoter-proximal pausing and release of Pol II has been identified as a major rate-limiting step for controlling the expression of many metazoan genes and plays a critical role in cell growth, renewal, and differentiation (Levine, 2011; Zhou et al., 2012).

Shortly after initiation, Pol II is paused at a promoter-proximal region by negative elongation factors NELF and DSIF, resulting in a short nascent transcript ~12 nts in length. Promoter-proximately paused Pol II is the major form of Pol II found on metazoan chromosomes and is poised for entry into productive elongation. The positive transcription elongation factor-b (P-TEFb) is the major factor required to overcome this restriction. P-TEFb, composed of cyclin-dependent kinase 9 (CDK9) and cyclin T1, phosphorylates and thereby antagonizes the inhibitory actions of NELF and DSIF, triggering the release of Pol II from promoter-proximal pausing. In addition to this, P-TEFb also phosphorylates the C-terminal domain of the largest subunit of Pol II, which then serves as a platform for assembling key transcription and RNA-processing factors that promote transcriptional elongation, cotranscriptional processing of pre-mRNA, and lastly termination (Zhou et al., 2012).

Under normal growth conditions, up to 90% of cellular P-TEFb is sequestered in an inactive complex called the 7SK RNP (Yang et al., 2001). Within this complex, a noncoding RNA, namely 7SK snRNA, functions as a scaffold and mediates the interaction of P-TEFb with HEXIM1 or -2 and thus inhibits CDK9’s kinase activity (Yik et al., 2003). 7SK snRNA, transcribed by RNA Pol III, is an abundant noncoding RNA and highly conserved in higher eukaryotes (Wassarman and Steitz, 1991). Although its levels remain relatively constant, several genome-wide studies suggested that 7SK was pseudouridylated (Kishore et al., 2013; Carlile et al., 2014). Indeed, site-specific and quantitative pseudouridylation assays demonstrated that up to 94% of 7SK snRNA in HeLa cells is pseudouridylated at residue U250. Although the guide RNA has not been identified, it is clear that the box H/ACA RNP machinery catalyzes this modification as the depletion of DKC1 significantly reduces 7SK snRNA pseudouridylation. In addition, 7SK snRNA pseudouridylation was demonstrated to play a critical role in regulating the formation of the 7SK-P-TEFb snRNP, as mutation of U250, or depletion of DKC1, reduced the binding of CDK9 and HEXIM to 7SK snRNA (Zhao et al., 2016).

The identification of a key role for pseudouridylation in 7SK snRNP stability has potential clinical relevance. For instance, one emerging strategy in curing human immunodeficiency virus (HIV) infection is to “shock,” or reactivate, the latent HIV reservoirs for their subsequent “kill” by Highly Active Anti-Retroviral Therapy (Richman et al., 2009; Deeks, 2012). Along this line, Zhao et al. demonstrated that reducing 7SK snRNA pseudouridylation destabilized the 7SK snRNP and activated Tat-dependent HIV-1 transcription. Furthermore, reduction in 7SK snRNA pseudouridylation in combination with latency reversal agents (LRAs) significantly increased the reversal of HIV latency, implicating the DKC1-box H/ACA RNP as a promising new target to eradicate latent viral reservoirs (Zhao et al., 2016). It will be interesting to determine whether the widely used chemotherapeutic agent 5-fluorouracil, which is an inhibitor of PUS enzymes, can act synergistically with current LRAs to further enhance the reversal of HIV latency.

## Function of Spliceosomal snRNA Pseudouridylation in Pre-mRNA Splicing

Most genes in eukaryotes are not transcribed in mature but rather as pre-mRNA, containing coding exons as well as noncoding introns. Therefore, further pre-mRNA splicing is needed to remove intronic sequences and assemble exons into mature mRNA (Green, 1986; Newman, 1994). Splicing is catalyzed by the spliceosome, a massively large complex consisting of snRNAs and numerous protein components (Wahl et al., 2009; Matera and Wang, 2014). There are five snRNAs within the major spliceosome—U1, U2, U4, U5, and U6 that participate in the splicing reaction as snRNP complexes.

**Figure 4:**
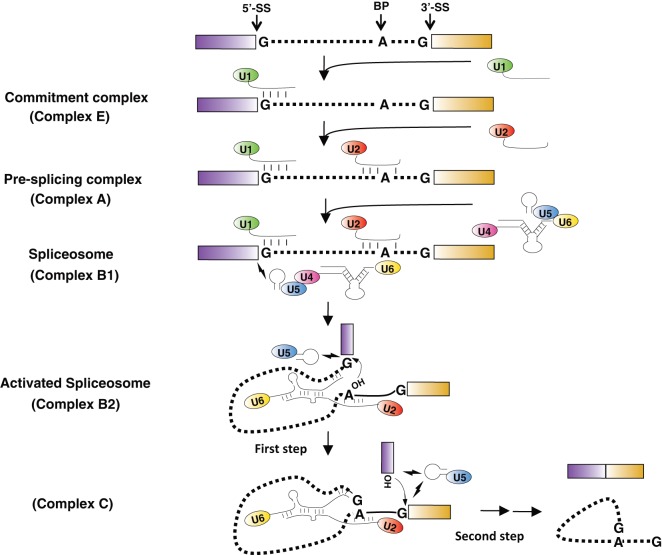
Major spliceosome assembly and pre-mRNA splicing. The dotted lines represent the intron, and the boxes are exons. The 5'-splice site (5'-SS), the 3'-splice site (3'-SS), and the branch point adenosine (BP) are indicated in the pre-mRNA. The conserved residues at the branch site and the 5'- and 3'-SS are shown. The thin lines are snRNAs with their names in the ellipses. The short thick lines between RNA strands represent Watson–Crick base-pairing interactions. The lightning symbols depict non-Watson–Crick base-pairing interactions. The 2'-OH groups of branch point adenosine and the cutoff 5' exon are pictured in the activated spliceosome. The small arrows near 2'-OH group indicate the nucleophilic chemical reactions.

Pre-mRNA splicing is initiated by the recognition of the 5'-splice site (5'-SS) by the U1 snRNP *via* complementary base-pairing interactions (Kramer et al., 1984; Bindereif and Green, 1987; Ruby and Abelson, 1988; Seraphin and Rosbash, 1989). The branch-site sequence is then engaged by the U2 snRNP *via* complementary base-pairing interactions, resulting in the bulging out of the branch point adenosine of the pre-mRNA and the formation of the spliceosomal complex A (Zhuang and Weiner, 1989; Michaud and Reed, 1991; Wassarman and Steitz, 1992). Subsequently, the tri-snRNP, a complex of U4 snRNP, U6 snRNP, and U5 snRNP, is recruited, creating a fully assembled spliceosome (complex B1). Following a series of RNA–RNA rearrangements, U1 and U4 snRNPs are destabilized and released, resulting in the formation of an active spliceosome (complex B2) (Sawa and Abelson, 1992; Lesser and Guthrie, 1993). This complex catalyzes the first step of pre-mRNA splicing in which the 2'-OH group of the bulged-out branch point adenosine nucleophilically attacks the phosphate at the 5'-SS. The result of the first-step reaction is the generation of a lariat 2/3 intermediate and a cutoff 5' exon intermediate. After additional conformational changes, complex B2 is converted to complex C, and the second step of splicing is catalyzed, resulting in the production of mature mRNA and lariat intron products. The U2, U5, and U6 snRNPs are recycled for new rounds of pre-mRNA splicing (Figure 4) (Burge et al., 1999).

**Figure 5:**
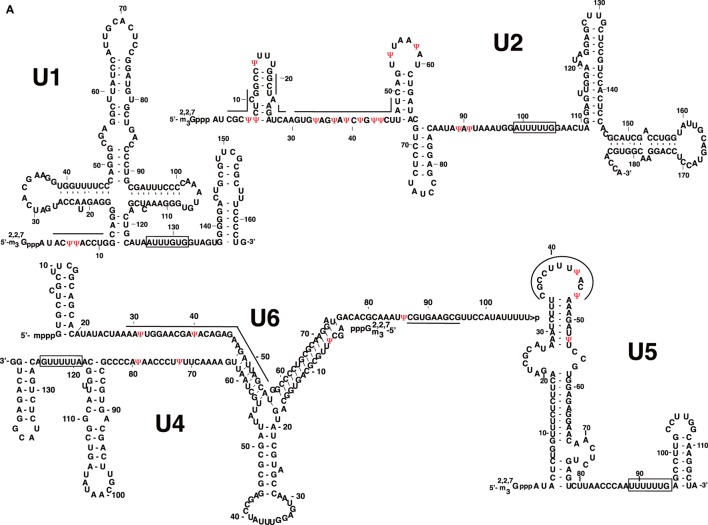

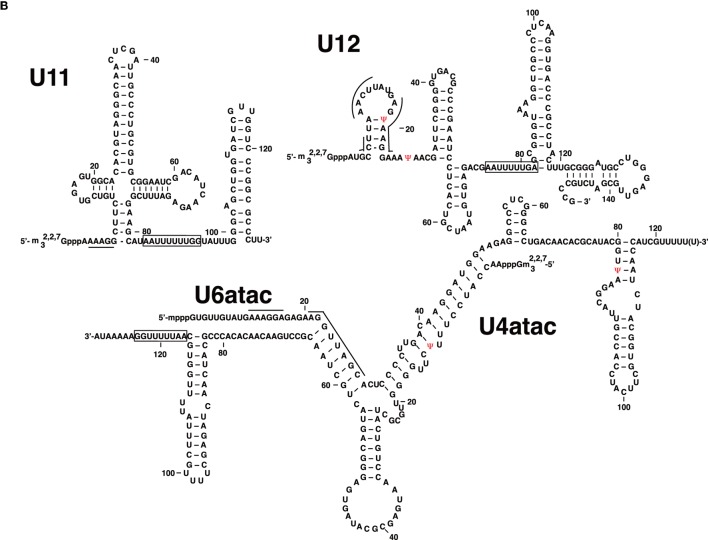
Pseudouridines in human spliceosomal small nuclear RNA (snRNAs). **(A)** Primary and secondary structures of human major spliceosomal snRNAs (U1, U2, U4, U5, and U6) are shown. **(B)** Primary and secondary structures of human minor spliceosomal snRNAs (U11, U12, U4atac, and U6atac) are shown. U5 snRNA is shared by both the major and minor spliceosomes. Pseudouridines (ψ) are in red. The thick lines indicate the nucleotides participating in RNA–RNA interactions or involved in catalysis during pre-mRNA splicing. The rectangles indicate the Sm-binding sites.

Interestingly, all of the major spliceosomal snRNAs are posttranscriptionally pseudouridylated (Figure 5A). U2 snRNA is the most extensively pseudouridylated snRNA, and unsurprisingly investigations into snRNA pseudouridylation have primarily focused on U2 snRNA. The functional study of U2 snRNA pseudouridylation was initiated in the early 1990s by Patton, who prevented U2 snRNA pseudouridylation in HeLa cell S100 and nuclear extracts by the incorporation of 5-fluorouridine (5-FU) (Patton, 1993a,b). Although 5-FU-substituted U2 snRNA was able to form a U2 snRNP, the snRNP was more vulnerable to dissociation by salt.

As experimental systems and assays developed, a more detailed description of the effects of U2 snRNA pseudouridylation on pre-mRNA splicing emerged. For example, Yu et al. demonstrated that while *in vitro*-transcribed U2 snRNA, which lacks modification, was unable to rescue a splicing defect in U2 snRNA-depleted oocytes, following prolonged reconstitution periods, U2 snRNA was pseudouridylated and able to reconstitute splicing activity. In addition, anti-snRNP immunoprecipitation coupled with glycerol-gradient sedimentation demonstrated that U2 snRNA lacking pseudouridine was unable to form functional 17 S U2 snRNP. Thus, a good correlation between modification status, U2 snRNP biogenesis, and pre-mRNA splicing was established. In addition, by creating chimeric U2 snRNAs between cellular-derived and *in vitro*-transcribed U2, Yu et al. demonstrated that the functionally important modifications primarily resided within the first 27 nts of U2 snRNA (Yu et al., 1998).

Pseudouridylation of residues within the branch-site recognition region is also functionally important for pre-mRNA splicing. Zhao and Yu demonstrated that pseudouridine residues within the branch-site recognition region of Xenopus U2 snRNA are required for U2 snRNP assembly and spliceosome assembly (Zhao and Yu, 2007). In addition, an NMR structure of yeast U2 snRNA:pre-mRNA branch-site helix demonstrated that ψ35 induces a dramatic structural alternation, which is required for the bulging out of the branch point adenosine and nucleophilic attack on the 5'-SS (Newby and Greenbaum, 2002). Consistent with this, a yeast knockout of PUS7, which catalyzes ψ35 of U2 snRNA, exhibited reduced fitness under conditions of high-salt media, or when in competition with a wild-type strain (Ma et al., 2003). The other pseudouridines within this region, ψ42 and ψ44, are also functionally relevant (Wu et al., 2016a). The deletion of both SNR81, responsible for ψ42, and PUS1, which catalyzes ψ44 formation, reduces the efficiency of pre-mRNA splicing, leading to growth defect. Further genetic and biochemical analyses demonstrated that U2 snRNA ψ42 and ψ44 facilitate the interaction with Prp5, a U2-dependent ATPase known to play an important role in monitoring U2-intron branch-site interactions at early stages of spliceosome assembly (Xu and Query, 2007). Furthermore, Prp5 has reduced ATPase activity on U2 snRNA lacking ψ42 and ψ44, suggesting that these modifications regulate Prp5 enzymatic activity. Collectively, these data indicate that pseudouridylation within the branch-site recognition region plays a role not only in the biogenesis of functional snRNP but also in spliceosome assembly by influencing the enzymatic activity of Prp5.

In contrast to U2 snRNA, pseudouridylations within the other spliceosomal U snRNAs have received much less attention. However, this is not to say that they have not been investigated. For instance, the functional importance of two ψs within the 5' end of U1 snRNA has been investigated. Adopting an *in vitro*-splicing system in which two 5'-SS are in competition with each other, Roca et al. suggested that U1 snRNA pseudouridylation participates in 5'-SS discrimination (Roca et al., 2005). In addition, Freund et al. demonstrated that a ψ–G base pair, between the U1 snRNA and the substrate pre-mRNA, respectively, stabilized the U1 snRNA interaction with the 5'-SS of HIV-1 RNA (Freund et al., 2003). Lastly, as described earlier, the inducible pseudouridylation of U6 snRNA at U28 during the yeast filamentous growth program is also functionally relevant, as shown by differential growth phenotypes that are dependent on its pseudouridylation (Basak and Query, 2014).

## Conclusion

Remarkable progress has been made toward elucidating the mechanism and function of RNA pseudouridylation in various cellular processes. However, a function for nc RNA pseudouridylation in transcriptional regulation has only just begun to emerge. Our limited understanding of the impact of pseudouridylation on transcription is partially due to the limited number of ncRNAs that were known to be pseudouridylated. However, recent efforts of transcriptome-wide mapping of RNA pseudouridylation have greatly expanded the catalog of known pseudouridylated RNAs and have identified novel modification sites within ncRNAs participating in transcriptional regulation. For instance, metastasis-associated lung adenocarcinoma transcript 1 (MALAT1), which is involved in the epigenetic modulation of gene expression, as well as alternative splicing, is pseudouridylated at two distinct sites (Carlile et al., 2014). How these modifications contribute to MALAT1 function remains unclear and certainly worth investigating. As more ψs in ncRNA transcriptional regulators are identified, a better understanding of how these ncRNAs impact eukaryotic mRNA transcription will be achieved. Since many of these ncRNAs are associated with diseases, determining how the functional impact of their pseudouridylation may open the door to novel therapeutic strategies.

Although the mechanism and function of snRNA pseudouridylation in splicing is considerably well studied, there are still many unanswered questions. For example, in contrast to U2 snRNA, the functional significance of pseudouridylation within U1, U4, U5, and U6 is not clear. In addition, within higher eukaryotes, there exists a second spliceosome, the minor spliceosome, which consists of U5, in addition to four distinct U snRNAs, U11, U12, U4atac, and U6atac, and all of them are pseudouridylated (Figure 5B). Interestingly, the positions of minor spliceosomal snRNA pseudouridylation are homologous to those within major spliceosomal snRNAs, suggesting their importance in minor intron splicing (Massenet and Branlant, 1999). Detailed functional analysis of these pseudouridylations is required if we seek to understand the mechanism of minor spliceosome biogenesis and minor intron splicing. In addition, these studies may provide a better understanding of how introns are selectively recognized by the two distinct spliceosomes.

## Author Contributions

All authors contributed to writing the manuscript.

## Conflict of Interest Statement

The authors declare that the research was conducted in the absence of any commercial or financial relationships that could be construed as a potential conflict of interest.
